# Roles of TRPM channels in glioma

**DOI:** 10.1080/15384047.2024.2338955

**Published:** 2024-04-29

**Authors:** Zhigang Chen, Han Xie, Jun Liu, JiaJia Zhao, Ruixiang Huang, Yufei Xiang, Haoyuan Wu, Dasheng Tian, Erbao Bian, Zhang Xiong

**Affiliations:** a Department of Neurosurgery, The Translational Research Institute for Neurological Disorders, The First Affiliated Hospital (Yijishan Hospital), Wannan Medical College, Wuhu, P. R. China; b Department of Neurosurgery, The Second Affiliated Hospital of Anhui Medical University, Anhui Medical University, Hefei, China; c Department of Orthopaedics, The Second Affiliated Hospital of Anhui Medical University, Hefei, China

**Keywords:** TRPM, glioma, function, signaling, therapy, target

## Abstract

Gliomas are the most common type of primary brain tumor. Despite advances in treatment, it remains one of the most aggressive and deadly tumor of the central nervous system (CNS). Gliomas are characterized by high malignancy, heterogeneity, invasiveness, and high resistance to radiotherapy and chemotherapy. It is urgent to find potential new molecular targets for glioma. The TRPM channels consist of TRPM1-TPRM8 and play a role in many cellular functions, including proliferation, migration, invasion, angiogenesis, etc. More and more studies have shown that TRPM channels can be used as new therapeutic targets for glioma. In this review, we first introduce the structure, activation patterns, and physiological functions of TRPM channels. Additionally, the pathological mechanism of glioma mediated by TRPM2, 3, 7, and 8 and the related signaling pathways are described. Finally, we discuss the therapeutic potential of targeting TRPM for glioma.

## KEY POINTS

•TRPM channels are widely expressed in the human body and play an important role in gliomas.

• Abnormal expression of TRPM2, 3, 7, and 8 channels in gliomas is associated with disease severity and prognosis.

•TRPM2, 3, 7, and 8 channels are effective targets in glioma.

## 1. Introduction

An ion channel is a protein consisting of pores that can regulate and passively ion flux through a biofilm (as determined by an electrochemical gradient).^1^ As far, there are more than 400 known ion channels, which belong to different families and are involved in a wide range of physiological processes.^2^ Meanwhile, abnormal expression of ion channels is closely related to many diseases, including cancer, hypertension, inflammatory pain, diabetes, and neurodegenerative diseases, suggesting that they may be fascinating therapeutic targets.^3–5^

TRP channels are transmembrane ion channels that allow the nonselective passage of cations through the cell membrane.^6^ So far, more than 30 members of the TRP channel family have been cloned in mammals. These channels span a range of conformational states from closed, open, and partially open.^7^ Since 2017, significant progress has been made in studying the structural biology of the TRPM subfamily using cryo-electron microscopy.^8^ There are four independent TRPM ion channels in which the cryo-EM structures have been solved.^9^ The TRPM channels represent one of the largest and most diverse subfamilies of the TRP superfamilies and are expressed in almost all cell types.^10^ In recent years, the TRPM subfamily has attracted considerable attention due to its involvement in several physiological and pathological processes, including temperature sensing,^11^ cancer progression,^12^ vascular development,^13^ neurological diseases,^14^ endothelial dysfunction^15^ and numerous others.^16,17^ This makes TRPM channels a fascinating group of ion channels with a high biomedical projection.^2^ As such, the TRPM channels have inspired scientists’ enthusiasm for functional and structural studies.

Over the past decade, TRPM channels have been shown to play a critical role in tumorigenesis, including gliomas.^18–20^ Gliomas, including anaplastic astrocytoma and glioblastoma, are the most common types of primary brain tumors.^21^ Chemotherapy is a major treatment for glioma besides surgery. Due to the lack of drugs with blood-brain barrier permeability, the clinical efficacy of glioma is greatly limited.^22^ Therefore, there is a need to search for new targets and drugs that are effective against glioblastoma. Among them, the TRPM channel represents a new intervention target. As epithelial cells evolve from normal to tumor growth, genetic changes can affect TRPM expression or lead to changes in TRPM channel activity.^23^ The TRPM channel is expressed in both normal and tumor tissues of the brain, which can regulate glioma growth and improve migration and invasion ability.^24^

Research confirmed that TRPM2 and TRPM3 have anti-tumor effects, while TRPM7 and TRPM8 may be related to glioma malignancy (such as proliferation).^25^ For example, Ca2+ permeates TRPM2 channels to enhance H2O2-induced human A172 glioma cell death.^26^ TRPM7 suppression significantly inhibited the growth, proliferation, migration and invasion of A172 cells.^27^ In addition, the expression level of TRPM8 was significantly correlated with the aggressiveness of glioma cells.^28^ These make the TRPM channels a potential prognostic indicator and therapeutic target.

This article reviews the research progress of TRPM subfamily members in recent years, focusing on the pathological function, related signaling pathways, and application of related drugs of the TRPM channels in glioma.

## 2. The TRP superfamily

The TRP channel superfamily was first discovered in mutant Drosophila melanogaster in 1969.^29,30^ Minke et al. named it TRP according to its electrophysiological phenotype.^31^ Electroretinogram measurements revealed a transient response to a steady light stimulus in vision-impaired mutant flies compared to wild flies, rather than the sustained voltage response typical of wild flies.^32,33^ In 1989, Montell and Rubin discovered the TRP gene and described its molecular characteristics.^34^ In 1992, the first homolog Trpl was found. Then, in 1993, the TRP family was identified as a new superfamily of ion channels, and its role in Ca2+ permeability was identified.^35^ In 1995, the first human homolog, transient receptor potential channel associated protein 1 (TRPC1), was identified.^36^ Since then, TRP channels have been found to present in large amounts in multicellular organisms, including but not limited to zebrafish,^37^ humans,^38^ mice,^39^ and worms.^40^ Unlike other ion channels, it is defined by the membrane’s sequence homology and topological structure.^41,42^ The structure of TRP has been highly conserved during the evolution from nematodes to flies and humans.^43^ Based on the amino acid homology of TRP channels, they were further divided into seven subfamilies: vanilloid (TRPV1–6), Canonical (TRPC1–7), melastatin (TRPM1–8), Mucolipin (TRPML1–3), Polycystin (TRPP1–3), Anchor protein Type (Ankyrin, TRPA1), TRPNs and TRPA1s.^44^ The subfamilies showed remarkable differences in the structure and sequence homology, resulting in different physiological functions.^29,45^ Some TRPs (e.g., TRPC1 and TRPM7) are widely expressed, while others are restricted to specific organs (e.g., TRPV5 is only expressed in the human kidney).^46^ Many diseases are associated with TRP channel dysfunction, such as surface-level abnormalities, cell translocation, or mutation, suggesting its essential role in homeostasis and disease in vivo.^47^

TRP exists in the plasma membrane as six transmembrane domain (S1-S6) polypeptide subunits. It requires four subunits to assemble homologous or heterotypic oligomeric pores into functional channels.^48^ S4 corresponds to a region similar to a voltage sensor that senses changes in intracellular ion concentrations.^2^ Channel hole by the S5 and S6 subunits between alpha spiral ring formation.^49^ Differences in subunit composition may lead to differences in the biological characteristics of TRP channels.^50^ Furthermore, TRP channels can permeate both monovalent and divalent ions, with some notable exceptions. TRPM4 and TRPM5 only pass through monovalent cations, while TRPV5 and TRPV6 show unusually high selectivity for Ca2+.^51,52^

Since Ca2+ is both the primary charge carrier and the most important second messenger, the TRP superfamily naturally becomes fundamental to the survival and function of cells.^53^ Moreover, TRP channels in muscle contraction, ion homeostasis, bone remodeling, and vasomotor control also play an essential role.^54^ TRP channels have many binding partners, mainly in channel regulation.^55^ When activated, TRP channels allow cations to flow in, leading to cell depolarization.^56,57^ Different cations have different permeability between channels, leading to various cellular effects.^58^ For example, Ca2+ and Mg2+ ions cross the cell membrane through TRP channels and play an essential role in various physiological processes, including cell proliferation, gene transcription, muscle contraction, and cell death.^59^ Early in the disease, the release of various endogenous substances can affect TRP pathway function, leading to infection and disease progression (for example, the release of leukotriene B4 induces TRPV1 activation).^60^ One of the most critical pathological processes is the development of cancer cells. More and more evidence has shown that changes in TRP channel expression levels play a crucial role in the occurrence and development of tumors, including proliferation,^61^ migration,^62^ invasion,^63^ and other characteristics of glioma. Under the background of brain tumors, all TRP channels have been studied for their potential role in cancer progression or as therapeutic targets.

## 3. TRPM subfamily

**Figure 1.**
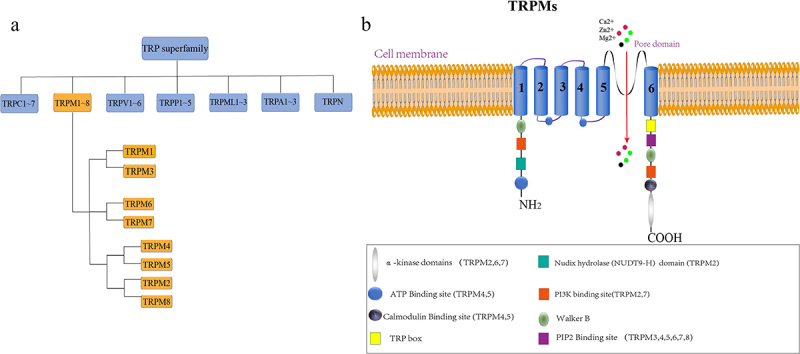
Members and general structure of the TRPM channels.

As mentioned, the TRPM channels represent one of the TRP superfamily’s largest and most diverse subfamilies.^64^ Since TRPM1 was first identified in 1989, much progress has been made in identifying new members and functions of the TRPM channels.^2^ TRPM subfamily consists of eight members, ranging from TRPM1 to TRPM8.^65^ These genes are widely expressed in the human body and involve numerous physiological and pathological features, particularly in ion homeostasis^29^ and cell development.^66^ TRPM channels promote intracellular Ca2 + signal in response to lipid, ion concentration, or second messenger to regulate these functions.^10^ Besides TRPM6 and TRPM7 being physically associated with bivalent on the membrane potential of selective, most of the channels are nonselective channels.^67^ Except for TRPM4 and TRPM5, other TRPM channels can be permeated by Ca2+, which are univalent cation channels activated by Ca2+ and functionally guide Na+ ions.^68^ Despite some shared structural features, members of the TRPM channels are less conserved than other members of the TRP family.^69^ According to sequence similarity, they were divided into four subgroups: 1) TRPM1 & TRPM3, 2) TRPM6 & TRPM7, 3) TRPM4 & TRPM5, 4) TRPM2 & TRPM8.^70^ Figure 1(a) summarizes their relationship.

## 4. Structure and domain organization of the TRPM channels

Due to the highly sequentially conservative nature of the TRPM subfamily, the overall structure of most TRPM members is similar.^71^ However, they also have some differences, which will be reviewed in detail from the following aspects: S1-S6 domain, N-terminal domain, and C-terminal domain, as shown in Figure 1(b). The pore domain on the cell membrane is connected to S1-S4 by the S4-S5 domain. The S1-S4 domain contains ligand-binding sites, such as the agonist Ca2+. In addition, the S1-S4 domains of K+ and Ca2+ channels contain voltage sensors. The S5 and S6 domains form ionic conducting pores with the p ring.^10^ As for the N-terminal, all TRPM channels have about 700 amino acids.^72^ As for the C-terminus, except for TRPM1 and TRPM2, all other TRPM members contain a highly conserved TRP box and phosphatidylinositol 4,5-bisphosphate (PIP2) binding site. However, there are some structural differences between TRPM1 and TRPM2. For example, TRPM1 and TRPM2 have the binding site of PIP2,^7^ but TRPM2 also has a NUDT9-H domain that can act as a binding site for ADP-ribose (ADPR).^73^ In addition, calmodulin (CAM) binding sites and ATP binding sites were also found at the TRPM4 and TRPM5 channels, and these channels could affect the channel’s Ca2+ sensitivity.^74^

Interestingly, the presence of C-terminal enzyme domains in 3 TRPM members, such as TRPM2, TRPM6, and TRPM7, is particularly notable, the only characteristic of all known ion channels to date. Specifically, TRPM2 possesses a domain at this location with a large amount of homology with NUDT9, a Nudix enzyme with ADP-nuclear hydrolase activity, while TRPM6 and TRPM7 also contain kinase domains and belong to atypical α-protein kinases.^75^

## 5. Activation mechanisms of the TRPM channels

**Figure 2.**
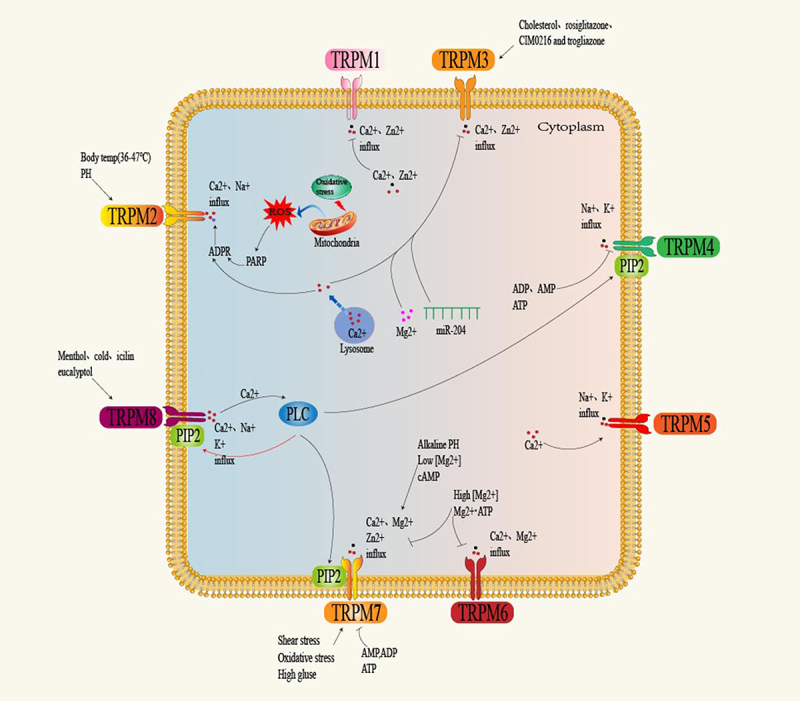
Activation mechanisms of the TRPM channels.

The TRPM channels are essential cellular receptors that can permeate Mg2+, Ca2+, Zn2+, and Na+ cations. As shown in Figure 2, different physical or chemical stimuli, such as temperature, osmolality, and PH, can activate TRPM channels.^76^

Although TRPM1 was first discovered, the mechanism of TRPM1 channel activation has yet to be fully elucidated. Studies have found that the change in ion concentration can also negatively impact channel activity. [Ca2+] cyt inhibits the increase of TRPM1 channels, and intracellular Zn2+ blocks the increase of TRPM1 channels.^77^

Because TRPM2 is widely distributed and has different physiological effects, its activation involves a variety of external stimuli. There is some controversy in the literature regarding TRPM2 activation, particularly nucleotide activation. ADPR generally has the highest affinity of TRPM2 receptor agonists (EC50, 10–80 μM).^78,79^ Other more controversial activators include nicotinate adenine dinucleotide (NAAD), cyclic ADPR (cADPR), nicotinamide adenine dinucleotide (NAD), and NAAD-phosphate (NAADP).^80^ Due to the low affinity of some of these activators, their direct role in TRPM2 activation has been controversial. However, most of them use ADPR as the activation mechanism. Recent evidence suggests that TRPM2 channel activation is related to its enzymatic domain.^81^ Extracellular signaling stimulates the production of ADPR in mitochondria by activating poly (ADPR) polymerase (PARP) or poly (ADPR) glycohydrolase (PARG), which subsequently binds to the C-terminal NUDT9-H domain of TRPM2 to activate the channel.^82,83^ Previous studies have found that cADPR and pyridine dinucleotides can activate TRPM2 or promote ADPR activation. However, when contaminated ADPR was purified with either nucleotide phosphatase or ADPR hydrolase, neither stimulated TRPM2 activation.^80^ These evidence suggests that ADPR is an activator of TRPM2. In addition to ADPR, increased intracellular Ca2+ positively modulates TRPM2.^84^ The initial calcium entry via ADPR-bound TRPM2 or a Ca2+ spark activation channel starts from cytosolic activity.^7^ Ca2±bound calmodulin then combined with the N-terminal IQ motif of TRPM2, providing positive feedback for activating the TRPM2 ion channel and increased Ca2+ influx.^85^ However, in the absence of internal or external Ca2+, ADPR is ineffective in activating TRPM2 channels.^86^ Furthermore, PIP2 has been found to affect the sensitivity of TRPM2 to Ca2+ activation.^82^ Other intracellular Ca2±dependent activators make TRPM2 sensitive to ADPR by interacting with CaM and can also splice ADPR-free TRPM2 in their isoforms.^78^ It has also been found that TRPM2 is sensitive to temperature, and the channel activity is inhibited by acidification.^87^ Finally, other external activators include ROS, tumor necrosis factor-α (TNFα), reactive nitrogen (RNS), Zn2+, Falcon A, amyloid beta peptide, and H2O2.^88^

TRPM3 channels can be activated by CIM0216, hypotonic solution, and pregnenolone sulfate (PS). When activated, it induces an increase in [Ca2+] cyt, leading to Ca2+/calmodulin regulation and subsequent activation of mitogen-activated protein kinases (MAPKs).^89^ It is also activated by plant-derived and synthetic compounds, including iodine, cholesterol, troglitazone, and rosiglitazone.^90^ In addition, changes in ion concentration can also affect channel activity. Several studies have found that [Ca2+] cyt and intracellular Mg2+ ions inhibit the activity of TRPM3 channels.^77,91^

Both TRPM4 and TRPM5 require an increase in intracellular Ca2+ levels for activation. Both are regulated by PIP2 but differ in their high sensitivity to Ca2+.^92^ TRPM4, identified as a homolog of MLSN (TRPM1), is highly expressed in the colon, prostate, kidney, and heart.^93^ The c-terminal CaM binding site of TRPM4 is critical for susceptibility to Ca2±dependent activation. For TRPM5, TRPM5 is 5 to 10 times more sensitive to Ca2+ than the TRPM4 channels.^2^ Initially identified as MTR1, TRPM5 was later reported to co-express with the taste-signaling molecule α-gustducin.^94^ Nevertheless, both TRPM4 and TRPM5 have a direct Ca2±binding site on the inner side of the cell in the S1-S4 domain. A recent study found that SOCE may be a source of Ca2+ supply, with TRPM4/5 mediated depolarization providing negative feedback for Ca2+ inflow through the SOCE channel.^95^ Furthermore, low micromolar AMP, ADP, and ATP inhibit TRPM4 but not TRPM5 and their activity is enhanced via phosphorylation of the protein kinase C (PKC)-dependent TRP domain.^10^

TRPM6 and its homology TRPM7 are activated when Mg2+ and Mg ATP levels are reduced in the cytoplasm.^96^ TRPM6 channels are inactive at basal levels of Mg2+ and lack sensitivity to Mg2+. However, TRPM6 is activated in the oligomeric form in response to the phosphorylation of TRPM7.^97^ As for TRPM7, other activators reported in the literature include Naltriben, prostaglandin E2, AMP, ADP, ATP, mechanical stimulation, etc.^98^ Notably, although Naltriben is highly similar in structure to other opioid receptor antagonists, such as NTX, no other opioid receptor antagonists activate TRPM7 channels.^99^

TRPM8 is probably the best-studied member of the TRPM channels. It is a cold receptor, which can be activated directly by hypothermia and chemical agonists such as menthol and modulated by critical molecules such as PIP2 and Ca2+.^100^ The sensitive region of PIP2 located in the C-terminal region is the determining factor of temperature sensitivity. For TRPM8 channels, PIP2 is a coolant agonist and a cofactor of the cryogenic activation channel.^101^ While PIP2 alone may be enough to activate TRPM8, the loss of base levels of PIP2 leads to channel desensitization. Moreover, activating TRPM8 by icilin requires basal cytoplasmic Ca2+ but does not require menthol activity.^102^

## 6. Molecular biological functions of the TRPM channels

In humans, TRPM1 is located on chromosome 15 and consists of 27 exons with a gene length of more than 58 KB.^103^ The TRPM1 gene has an mRNA transcription volume of about 5.4 KB and encodes the 1603 aa protein of approximately 182 kDa, expressed only in pigmentation cells of the skin and eyes.^104^ TRPM1 also has a short N-terminal isoform called MLSN1-S, which lacks all the transmembrane domains that produce 500aa proteins.^105^ It has been reported that MLSN1-S is located in the cytoplasm, while L-type (MLSN1-L) is located in the cell membrane.^20^

First reported by Nagamine et al., TRPM2 was identified as LTRPC and TRPC7.^106^ In CNS, TRPM2 is expressed in multiple regions, including the cortex, substantia nigra, spinal cord, hippocampus, and striatum.^107^ TRPM2 can also be detected in peripheral tissues such as the heart, liver, pancreas, and lungs.^108^ This widespread expression reflects the involvement of TRPM2 in various pathological and physiological processes. Moreover, the channel is expressed in mononuclear cell lines, including multiple cultures of macrophage cell lines, peripheral blood mononuclear cells, and neutrophils.^109^ TRPM2 channels exhibit calcium, cation channel, and ADP-ribose pyrophosphatase activity through C-terminal enzyme domains. The domain hydrolyzes ADP-ribose to ribose-phosphate and AMP but at a much lower efficiency than the known Nudix enzymes.^110^

The TRPM3 is expressed in the human brain, as well as in the kidneys of mice and humans.^111^ Differences in the transcript of the TRPM3 mRNA and protein products may lead to diverse molecular mechanisms, such as the induction of short variants by hypotonic solutions or the storage depletion of lengthy variants. This fact, coupled with the specific expression of TRPM3 in the kidney, suggests that this channel is involved in renal osmotic homeostasis. Due to alternative splicing, the TRPM3 gene produces many different mRNAs.^112^ Although Lee et al. cloned six variants from human kidneys, three transcripts of varying lengths were detected in the mouse brains.^113^ Different starting positions and C-terminus give TRPM3 transcripts a high degree of variability. In addition, TRPM3 ion channels function independently of TRPV1’s thermal sensor.^114^ TRPM4 and TRPM5 are calcium-activated sodium ion channels impermeable to calcium and mediate plasma membrane depolarization.^115^ The binding sites of CaM, PKC phosphorylation sites, and ATP in TRPM4 agree with the regulatory activity of calcium, AMP, ADP, ATP, and Ca2+ sensitivities in the channel.^20^ Furthermore, TRPM4 has ATP-binding sites that may inhibit TRPM4 by stabilizing channels in the apolipoprotein-like conformation.^116^ Selective expression of TRPM5 was initially reported in taste buds, suggesting that it may be involved in taste transduction.^117^ Subsequently, the immune reactivity of TRPM5 was found in the olecranon and nose. It suggested that TRPM5 is an inherent signaling component of the mammalian chemosensory organs.^118^ TRPM4 and TRPM5 are both voltage-gated and regulated by PIP2 and phospholipase C (PLC). Moreover, these two channels play a role in T lymphocyte secretion, cytokine secretion, and myogenic contraction of cerebral arteries.^119^

TRPM6 and TRPM7 are most closely related to TRPM1 and TRPM3. In fact, despite their different C-terminal domains, they share the same N-terminal sequence.^120^ Furthermore, these four channels are similar in the small cytoplasmic region, the putative channel, and the transmembrane region after the sixth transmembrane region. TRPM6 and TRPM7 are closely related, and both have a protein kinase domain at the C-terminus. This domain has been shown to form autophosphorylated dimers and phosphorylate protein substrates.^121^ TRPM6 and TRPM7 preferentially penetrate Mg2+ and Ca2+ but may be the most important for maintaining physiological Mg2+ homeostasis. In addition to a wide variety of divalent positive ions, TRPM7 channels can penetrate univalent ions, especially H +.^122^ Interestingly, TRPM6 has been found to interact specifically with the homolog TRPM7, leading to the formation of functional TRPM6/TRPM7 complexes in cell membranes. In the presence of Mg2+ and Mg2+·ATP, the binding of mTRPM6 and mTRPM7 results in the high intrinsic activity of mTRPM6/7 at the physiological level.^123^

TRPM8 is a homotetramer composed of 130 kDa subunits. It is located on chromosome 2 and contains 102 kilobases and 27 exons.^124^ Multiple transcription factor binding sites on its promoter are famous, such as MYC, NKX3–1, and USF1. TRPM8 forms 11 mRNA isomers by alternative splicing. Among them, the isomers SM8α and SM8β are detected in the prostate and regulate the production of full-length proteins.^125^ TRPM8 protein contains 1104 AA residues, which is the main conserved region of the TRP family. There are eight glycosylation sites and one immune antigen epitope. TRPM8 is expressed in a variety of cells, but mainly in the dorsal root and trigeminal ganglion of the peripheral nervous system.^126^ In the cell, this channel shows the location of the plasma membrane and membrane raft. However, TRPM8 has also been found in the mitochondria-associated endoplasmic reticulum (ER) membranes (MAMs) in prostate cancer cells.^127^ Thus, TRPM8 channels may have a role far beyond their role in somatosensory neurons. In addition, the protein is also found in other tissues, such as the bladder, vascular smooth muscle, the liver, and lungs.^89^

## 7. Pathological functions of the TRPM channels in glioma

Most TRPM members are extensively involved in developing malignant tumors.^18^ Among them, TRPM2, 3, 7, and 8 are closely related to the occurrence and development of glioma. TRPM2 and TRPM3 have antitumor effects, and TRPM7 and TRPM8 may be involved in the malignant transformation of glioma.^63^ Therefore, this chapter will focus on the relevant pathological functions of TRPM2, 3, 7, and 8 in glioma.

### 7.1. TRPM2

Oxidative stress is a prominent feature of many pathologies, and a common mechanism is to induce cell death by disrupting intracellular ion homeostasis.^73^ Recent evidence suggests that TRPM2 channels are susceptible to oxidative stress activation and play a vital part in oxidative stress-induced cell death, including gliomas.^128^ For example, introducing TRPM2 into human glioblastoma multiforme (GBM) cells enhanced the hydrogen peroxide-induced apoptosis. Interestingly, TRPM2 has also been found to induce autophagy. In most cases, TRPM2, located on the lysosome membrane, causes cell death through autophagy. Moreover, studies have shown that TRPM2 may also induce autophagy by acting on the early steps of autophagy in cancer.^129^ Apoptosis and autophagy are common forms of cytotoxicity induced by anticancer drugs. For example, the effect of selenium (Se) on docetaxel-resistant GBM cells may be to activate TRPM2 through oxidative stress and enhance the apoptotic effect of DTX.^19^

In addition to inducing apoptosis and autophagy, TRPM2 may play a vital role in glioma progression since TRPM2 channels are also found in cerebral blood vessels.^130^ Furthermore, abnormal TRPM2 function appears to be associated with psychiatric and neurological disorders, such as stress depression, epilepsy, ischemic brain damage, and glioma invasion.^131^ Moreover, TRPM2-S and TRPM2-AS are also closely associated with malignant tumors such as gliomas.^132^ TRPM2-S is an isomer of TRPM2, which activates the ERAD system to ubiquitinate TRPM2 and inhibit TRPM2 activity by interacting with TRPM2 located at the cytoplasmic membrane.^133^ TRPM2-AS has recently been identified as an antisense lncRNA located at chromosome 21q22.3.^134^ It is an oncogene for prostate cancer, hepatocellular carcinoma, and glioma.^135,136^ In gliomas, overexpression of TRPM2-AS is closely related to cell proliferation, invasion, and migration. The same phenomenon was found when TRPM2-AS was down-regulated by siRNA.^132^ In conclusion, TRPM2, TRPM2-S, and TRPM2-AS are closely related to the occurrence and development of glioma.

### 7.2. TRPM3

TRPM3 is widely distributed in the body, especially in the brain, and the dysregulation of its expression is closely related to glioma. Still, there are few reports on the pathological function of TRPM3 in glioma. For example, TRPM3 expression was upregulated in glioma. TRPM3 may play a vital role as a tumor suppressor gene in glioma. Its loss of expression is a marker of poor prognosis in GBM patients because TRPM3 expression is significantly down-regulated with increased glioma grade.^63^ Furthermore, DNA methylation of the TRPM3 promoter was positively correlated with glioma dryness and migration.^137^ Moreover, TRPM3 has also been closely related to glioma growth and autophagy,^138^ but its mechanism needs further research. In conclusion, methylation of the TRPM3 is involved in the malignant phenotype of glioma.

### 7.3. TRPM7

TRPM7 is widely expressed throughout the body and is closely associated with many central nervous system lesions, including glioma.^121,139^ For example, one study has found that TRPM7 channels are closely related to the angiogenesis of glioma.^140^ Another study demonstrated TRPM7-dependent CLIC1 vesicles translocated to microvascular endothelial cells in glioma. It suggested that TRPM7 channels may promote GBM angiogenesis.^141^ As mentioned above, TRPM7 is a bifunctional protein containing an ion channel and kinase structure.^142^ Although TRPM7 mediates cell migration, invasion, and proliferation in glioma, migration and invasion ability are mainly increased by α-kinase domains, while proliferative capacity is increased by ion channel activity.^24,61^ These lines of evidence suggest that TRPM7 channels and kinase domains are closely related to glioma proliferation, migration, and invasion.

### 7.4. TRPM8

Various diseases, such as chronic cough, cancer, migraines, and irritable bowel syndrome, have been linked to TRPM8 pathology. Early studies found that in more than 20 TRP channels, compared with normal brain tissue, TRPM8 mRNA expression in GBM was upregulated to the highest level, which was closely related to the migration, invasion, and proliferation of glioma.^143^ TRPM8 suppression or knockdown has been shown to disrupt the trigger of apoptotic cell death, cell cycle, cloning survival, and impair DNA repair.^144^ TRPM8 channels may also regulate cell proliferation by dynamically controlling the level of glioma resting potential, which may be a crucial cell cycle regulator. Furthermore, one study found two TRPM8 variants, with a more significant menthol-induced increase in Ca2+ influx observed in migrating cells than in non-migrating cells, possibly indicating that only migrating cells express full-length TRPM8 proteins in the plasma membrane.^63^ Nevertheless, these results must be treated with caution in future cancer treatment strategies, as TRPM8 has been found to have anti-migration activity against other cancers, such as prostate cancer.^145^ Interestingly, ionizing radiation can also activate BK channels to induce migration. The mechanism proved that ionizing radiation activates and upregulates TRPM8 in glioma,^63^ thus confirming the direct and indirect interaction in controlling glioma migration. Moreover, the increased invasion rate of glioma is also related to TRPM8 overexpression. In addition, HGF/SF is a multifunctional cell effector that can affect TRPM8-induced cell migration and Ca2+ homeostasis enhancement, meaning that TRPM8 is invasive and resistant. The evidence also indicates that TRPM8 may be enriched in HGF/SF and cMET, which is vital in malignant tumors, including GBM.^146^ Furthermore, TRPM8’s effect on GBM progression was far greater than its effect on cell migration and invasion. It also dramatically affects other decisive processes such as cell survival, cell cycle, and radiation resistance.^144^ In summary, the above evidence proves that the pathological function of TRPM8 is closely related to the migration, invasion, and proliferation of glioma.

## 8. Signaling pathways of the TRPM channels in glioma

**Figure 3.**
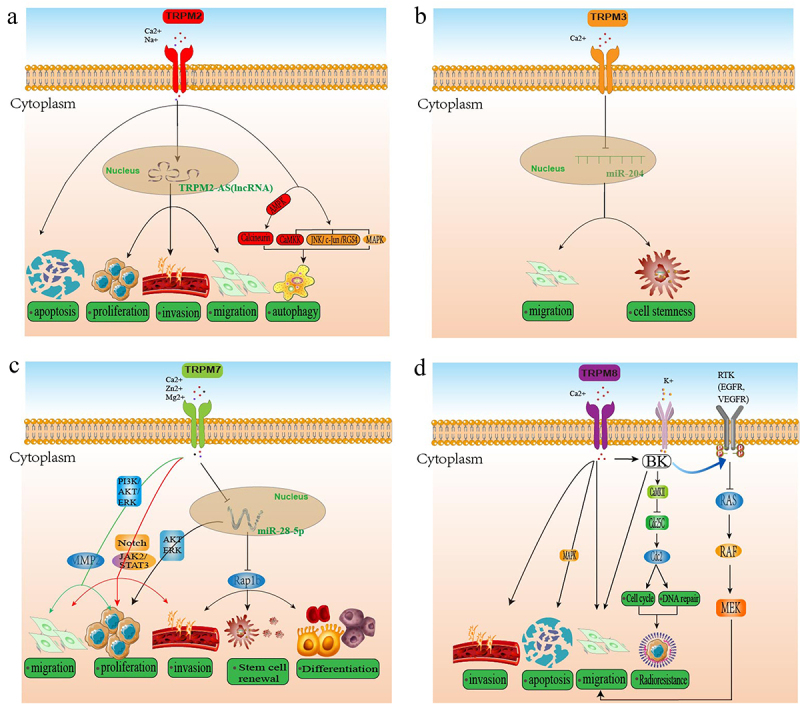
The main TRPMs-mediated mechanisms and signaling pathways associated with the development and progression of glioma.

Four members of the TRPM channels, TRPM2, 3, 7, and 8, are widely involved in the signal transduction process of glioma, such as cell-death-related signaling pathways, RTK/RAS/PI3K signaling pathways, MAPK/ERK signaling pathways, JAK2/STAT3/Notch signaling pathway, miR-28-5p/Rap1b axis, CaMKII, BK, and Kir4.1-K+ channels, etc. These signaling pathways are reviewed in detail below, as shown in Figure 3(a–d).

### 8.1. TRPM2-mediated cell death-related signaling pathways in glioma

Tumor cell death cannot be separated from various signaling pathways. TRPM2 is closely associated with cell death-related signaling pathways in glioma, especially apoptosis and autophagy.^20^ TRPM2-mediated apoptosis was induced mainly through the mitochondrial pathway. TRPM2 activation leads to increased intracellular Ca2+ concentration and oxidative stress, which promotes glioma cell death through apoptosis.^63^ Specifically, Oxidative stress is closely related to the increase of ROS. ROS increases NAD+/ADPR levels or induces oxidative stress, triggering TRPM2 activation. Subsequently, TRPM2 induces MPTP opening and mitochondrial damage through excess Ca2+ and Na+, leading to caspase-3-dependent apoptosis, apoptotic body formation, and nuclear condensation. Interestingly, mitochondrial fission may be one of the pathways through which mitochondria trigger TRPM2-mediated apoptosis. Previous studies have suggested that TRPM2 may initiate pancreatic cell mitochondrial apoptosis by mediating the lysosome Zn2+ layer.^73^ Nevertheless, the source of elevated Zn2+ needs to be clarified in the study, and we speculate that this mechanism is also related to glioma cell apoptosis.

Protein kinase C-α can induce the decoupling of spliceosome TRPM2-S and TRPM2 and activate cell apoptosis. For example, TRPM2-S induces apoptosis in liposarcoma by increasing ROS levels.^82^ So far, there have been no studies on glioma. In addition to inducing apoptosis, TRPM2 can induce autophagy through a mechanism that has been shown to activate a range of Ca2±dependent receptors and signaling cascades by altering cytoplasmic Ca2+ concentrations. Such as CaMKK, MAPK and JNK/c-Jun/RGS4.^132^ Moreover, lysosomal Ca2+ signaling was found to activate AMPK and trigger autophagy via calcineurin. In conclusion, these results suggest that TRPM2 mediates glioma cell death through apoptosis and autophagy signaling pathways related to cell death.

### 8.2. TRPM3 plays a protective role in glioma by targeting the miR-204

MicroRNAs (miRNAs) are endogenous, small non-coding RNAs that negatively regulate gene expression.^147^ As one of them, miR-204 originates from the 6th intron of the TRPM3 gene,^148^ while the TRPM3 promoter controls the expression of miR-204. miR-204 has been reported to act as a tumor suppressor gene in various malignancies,^149^ including glioma. For example, miR-204 exerts tumor-suppressive effects by promoting apoptosis, conferring cancer cell resistance to chemotherapy, inhibiting epithelial-to-mesenchymal transformation (EMT), and self-renewal of cancer stem cells (CSCs).^150^ Interestingly, studies have shown that miR-204 is substantially down-regulated due to hypermethylation of the TRPM3 promoter, which is related to enhanced cell stemness and migration.^137^ In addition, in nude mouse brain transplant tumors, miR-204 restoration consistently inhibited tumor invasion and improved animal survival.^63^ However, the role of TRPM3 and the regulatory function of miR-204 have been questioned. For example, upregulation of miR-204 expression in glioma leads to reduced ezrin levels through classical interaction with MRE (miRNA recognition element) in ezrin 3’ UTR.^151^ In conclusion, TRPM3 is closely related to the function of miR-204 and plays a protective role in glioma.

### 8.3. Extensive TRPM7-related signaling pathways in glioma

#### 8.3.1. TRPM7-related RTK/RAS/PI3K and MAPK/ERK signaling pathways in glioma

RTK/RAS/RAF/MEK/ERK signal cascade is essential in intercellular and intracellular communication and is a ubiquitous signal transduction pathway.^152^ It is often altered in disease and causes many human syndromes and conditions, including cancer.^153^ To date, RTK/RAS/PI3K and MAPK/ERK signaling pathways related to TRPM7 are closely associated with the migration, invasion, and proliferation of glioma, which are crucial for glioma progression. For example, one study found that growth factors such as EGF and VEGF regulate TRPM7 activity through the RTKS signaling pathway.^154^ Another study confirmed that TRPM7 enhances the MAPK/ERK and/or PI3K/Akt pathways in various cancers, including glioma.^155,156^ Furthermore, MAPK/ERK and PI3K/Akt are essential signaling pathways that show key roles in glioma migration, invasion, and proliferation. Interestingly, PI3K/Akt signaling hyperactivation occurs in approximately 45% of GBM patients.^157^ Inhibiting the PI3K/Akt signaling pathway can inhibit glioblastoma growth. Moreover, Naltriben has been shown to enhance GBM migration and invasion by inducing TRPM7-like currents through Ca2 + influx, associated with increased activation of MAPK/ERK-related proteins.^99^ Interestingly, Naltriben also enhanced TRPM7 channel activity, possibly by upregulating the MAPK/ERK signaling pathway and MMP-2 protein expression to enhance GBM migration and invasion. Besides Naltriben, the anti-migration and anti-proliferation effects of xylitone B on glioma are also related to Ras/MEK/MAPK and PI3K/Akt signaling pathways regulated by TRPM7.^121^

PI3K/Akt and MAPK/ERK signaling pathways are regulated by PLC.^121^ Therefore, TRPM7-related PLC isoenzymes may interact with the TRPM7α-serine/threonine protein kinase domain. Further research is needed to confirm these hypotheses. Interestingly, patients did not respond satisfactorily to treatment that inhibited only one of them. These evidence suggests a possible interaction between the MAPK/ERK pathway and the PI3K/Akt pathway, and a more effective strategy for treating GBM would be to target TRPM7.^99^

#### 8.3.2. TRPM7-related Notch and/or JAK2/STAT3 signaling pathway, and miR-28-5p/Rap1b axis in glioma

As a traditional inflammatory pathway, the JAK/STAT signaling pathway has also been found to promote the occurrence and development of cancer.^158^ It involves various tumorigenic functions, including replication, anti-apoptosis, angiogenesis, and immunosuppression.^159^ Abnormal activation of this pathway has been found in tumors, including gliomas, which may contribute to tumor development.^160^ TRPM7 can enhance malignant phenotype in glioma by upregulating Notch and JAK2/STAT3 signaling pathways.^140^ Furthermore, STAT3 binds the aldehyde dehydrogenase 1 (ALDH1) promoter, which is functionally involved in cell differentiation, self-protection, proliferation, and expansion, which may imply that TRPM7 is not only involved in migration, invasion, and proliferation but also glioma stem cell (GSC) differentiation and renewal.^161^ Moreover, the expression of TRPM7 was associated with the decrease of miR-28-5p. As previously mentioned, miR-28-5p also is a tumor suppressor whose downregulation leads to significantly increased proliferation and invasion of glioma.^61^ In the target of miR-28-5p, the expression of Rap1b in GBM was positively correlated with TRPM7 and negatively correlated with miR-28-5p. In fact, TRPM7 may also modulate the Notch pathway, recently implicated in integrin-mediated cell adhesion and Rap1b signaling.^63^

### 8.4. TRPM8 is involved in the MAPK and Ca2+/calmodulin-dependent protein kinase II (CaMKII) signaling pathways as well as BK and Kir4.1-K+channels of glioma

As mentioned before, MAPK signaling pathways related to TRPM8 are closely related to the malignant phenotype of glioma, which also shows a vital role in the cell death of glioma. For example, one study found that MAPK signaling is involved in TRPM8-mediated apoptosis of GBM cells,^162^ and another study confirmed that inhibition of the MAPK signaling pathway leads to growth arrest and stimulates apoptosis of GBM cells.^163^ Furthermore, TRPM8 interferes with cell cycle control via Cdc2, CaMKII, and cdc25C.^144^ More specifically, TRPM8-mediated Ca2+ entry increases CaMKII activity by activating the BK channels and then inhibits the Cdc2 subunit of the mitotic promoter by inhibiting Cdc25C phosphatase phosphorylation. Interestingly, TRPM8 channels also regulate GBM cell migration by activating K+ membrane ion channels (BK channels) activated by large conductance Ca2+.^164^ In addition, TRPM8 activation can also increase Kir4.1-K+ channels. Similar to the BK channels, Kir4.1 is a K+ channel with altered expression in the glioma.^165^ Its expression was negatively related to glioma migration and invasion behavior.^166^ In a word, TRPM8 is closely related to glioma signaling pathways, including the MAPK signaling pathway and CaMKII, BK, and Kir4.1-K+ channels.

## 9. TRPM channels drug application in glioma

**Figure 4.**
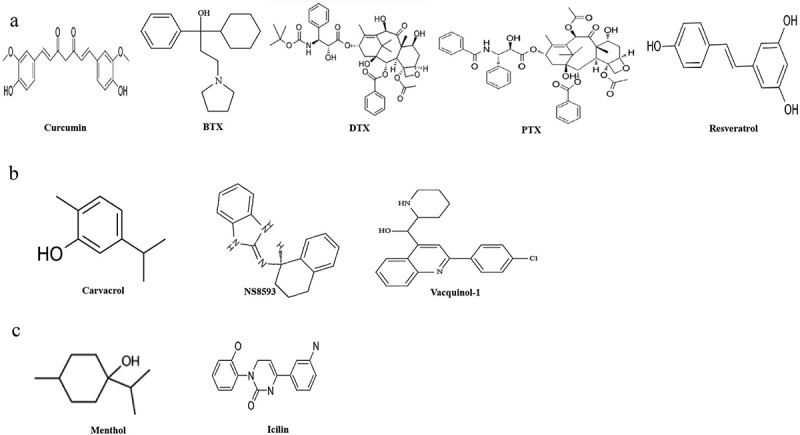
Structure of chemical agonists and antagonists of the TRPM channels in glioma.

Ion channels are widely expressed in almost all living cells and are the third largest drug target after enzymes and receptors. Considering the enormous contribution of altered expression of the TRPM channels to glioma formation and progression, they may be promising novel therapeutic targets. Figure 4(a–c) and Table 1 show the structure of commonly used TRPM channel drugs.

**Table 1.** Chemical inhibitors and activators of the TRPM family in glioma.

TRPM channels	Compound	Inhibitors and activators	IC50 (μM)	Defining structure	Chemical structure	Assay type	Reference
TRPM2	curcumin	inhibitor	4.8	Nudix box motif	Figure 3(a)	Electrophysiology	^157,158^
	BTX-A	activator	n.d		Figure 3(a)	Calcium flux	^158^
	DTX	activator	n.d		Figure 3(a)	Electrophysiology	^159^
	PTX	activator	0.176		Figure 3(a)	Electrophysiology	^160^
	resveratrol	activator	6.7		Figure 3(a)	Electrophysiology	^160,161^
TRPM3	n.d.	n.d	n.d	n.d	n.d	n.d	n.d
TRPM7	carvacrol	inhibitor	306	Serine/ threonine kinase domain	Figure 3(b)	Electrophysiology	^112^
	NS8593	inhibitor	1.6		Figure 3(b)	Electrophysiology	^166^
	VQ-1	inhibitor	n.d		Figure 3(b)	Electrophysiology	^165^
TRPM8	menthol	activator	11.8	n.d	Figure 3(c)	Electrophysiology	^55,167^
	icilin	activator	0.058		Figure 3(c)	Electrophysiology	^57,94^

Some data show that TRPM2 could be a good candidate for gene therapy. Combining TRPM2 with γ-radiation or chemotherapy drugs can improve the efficacy of GBM treatment. Moreover, TRPM2 promotes cancer cell death, including glioma.^73^ Next, curcumin, BTX-A, DTX, paclitaxel and resveratrol will be reviewed one by one. Previously, many studies have confirmed that the cytotoxic effect of curcumin on glioma cells is closely related to the induction of cell apoptosis and autophagy.^168^ The combination of curcumin and cisplatin suggested that curcumin enhanced cisplatin-induced death of human laryngeal squamous cell carcinoma (LSCC) cells through TRPM2.^169^ Cisplatin is a taxane widely used in glioma, breast cancer, and prostate cancer chemotherapy. Therefore, curcumin can enhance the efficacy in the case of cisplatin resistance, and it is an excellent drug combination for the treatment of glioma. In glioblastoma, it has been confirmed that BTX-A shows a vital protective role in glioma cell proliferation by stimulating cell apoptosis. Its molecular mechanism may be the up-regulation of the mitochondrial DRIA-related oxidative stress pathway and the activation of TRPM2.^167^ Furthermore, selenium (Se) tested in docetaxel (DTX) resistant GBM cells may enhance the apoptotic effects of DTX by activating TRPM2 through oxidative stress. More specifically, selenium activates TRPM2-mediated Ca2+ influx by stimulating the production of oxidative stress, thereby enhancing the same Ca2±dependent apoptotic pathway induced by DTX. Interestingly, the cytotoxic effects of DTX may also arise from Ca2+ influx into cells and the formation of excessive mitochondrial ROS, which leads to DNA damage and subsequent apoptosis by triggering the overactivation of PARP. In addition, the supplementation of selenium can alleviate the side effects of docetaxel.^19^ Recent studies have demonstrated the benefits of combined treatment with paclitaxel and resveratrol. Resveratrol was shown to enhance the efficacy of paclitaxel, particularly against paclitaxel-resistant breast cancer cells.^170^ When it comes to brain cancer, there are all kinds of findings. Ozturk et al. studied the combined effect of paclitaxel and resveratrol on GBM cells. The results showed that TRPM2 was involved in the mechanism by which resveratrol enhanced the effect of paclitaxel.^171^ Paclitaxel and resveratrol further increased apoptosis by increasing intracellular ROS levels and inducing mitochondrial dysfunction, suggesting that TRPM2 plays a role in regulating apoptosis in GBM cells. In conclusion, targeting TRPM2 may be a promising therapeutic approach, and researchers should actively explore its characteristics and mechanism as a therapeutic target for glioma.

Studies have found that the central pore is essential in drug transport.^172^ In the TRP family, TRPV2 and TRPM3-related drugs were found to be closely related to the central pore. For example, one study found that TRPV2 channels act as “drug carriers” in cancer treatment. TRPM3 promotes chemotherapeutic drugs’ absorption through the central pore, thereby improving the efficacy of cancer therapy.^173^ A similar central pore is present in TRPM3, as mentioned earlier. Other evidence is that the TRPM channels can act as a “drug carrier” due to the penetration of chemotherapy agents into cells through the pore domain. For example, activating TRPM3 channels may allow intracellular uptake of nonspecific chemotherapy agents by altering pore helical conformation.^57^ In this context, future studies should explore whether TRPM3 can promote chemotherapeutic drug absorption via the central pore pathway.

Tumor recurrence is attributed to migration, invasion, and resistance to therapy,^174^ and pharmacological inhibition of TRPM7 may be a novel therapy in glioma. It was also found that some TRPM7 channels, which interfere with cell division pathways, may be promising targets for drugs, making tumors more amenable to resection and local radiotherapy. For example, carvacrol affects glioma migration, invasion, and proliferation by inhibiting TRPM7 and regulating the G0-G1 cell cycle.^175^

Increasing evidence suggests that increased activation of TRPM7-mediated MAPK/ERK and PI3K/AKT signaling pathways are critical components of tumorigenesis and other signature cancer features. Like xyloketal B, carvacrol has been reported to induce xykeob-like effects in glioma by inhibiting MAPK/ERK and PI3K/AKT signaling pathways.^121^ In parallel experiments, it was found that carvacrol could reduce expression levels of p-Akt and p-ERK1/2, the cell viability, migration, and proliferation in GBM cells. Interestingly, Vacquinol-1 (VQ-1)-induced multitype glioblastoma cell death was in contrast to exogenous ATP-induced TRPM7 activity.^176^ The study of TRPM7 and its specific pharmacological inhibition confirmed that inhibition of AKT or ERK inhibitors is crucial for controlling GBM growth and invasion. Moreover, Siddiqui et al. used NS8593 to demonstrate that TRPM7 inhibits the migration and invasion properties of microglial cells.^177^ TRPM7 is a negative regulator of the angiogenic process.^61^ It suggests that specific TRPM7 activators can target both vascularization and cancer progression. Future studies should consider specific activators of TRPM7, such as Mibefradil, to be valuable for further investigation.

The low specificity of some TRPM8 agonists may have higher efficacy by triggering multiple channels simultaneously. For example, activating TRPM8 channels with menthol and icilin has increased the opening probability of individual BK channels.^63^ This channel mediates ion flux through the plasma membrane and supports cell contractile-driven cell migration.^178^ Furthermore, the overexpression of BK channels was found in glioma. Pharmacological inhibition of the channels was confirmed to eliminate cell migration and menthol-stimulated intracellular Ca2+ influx.^179^ Furthermore, ionizing radiation, which induces migration through Ca2+ -mediated activation of BK channels, has been shown to activate and upregulate TRPM8-mediated Ca2+ influx in glioma cells,^144^ thus confirming the direct interplay of the two channels in controlling glioma migration. Moreover, another study showed that TRPM8 agonists such as menthol could increase Kir4.1-mediated cell membrane conductivity in U251 GBM cells.^180^ Therefore, all the above evidence proves that the TRPM8 channels are a promising candidate for targeted glioma therapy.

## 10. Conclusions and Perspectives

With the deepening of the understanding of TRPM channels, it has been found that TRPM channels are widely involved in the physiological and pathological processes of the human body, such as temperature regulation, immune response, insulin secretion, and cold or osmotic regulation. In addition, the TRPM channels are closely related to the occurrence and development of tumors. There is increasing evidence that TRPM channels may be promising targets for cancer therapy. Studies of different TRPM members have advanced the understanding of the complexity of the TRPM channels. Since most TRPM structures are closed, much remains to be learned about the “control” of channel activation.

Here, we conclude that TRPM2, TRPM3, TRPM7, and TRPM8 isoforms are promising targets in glioma, with multiple pieces of evidence reporting their overexpression in cancer and demonstrating the requirements in cancer cell growth, survival, migration, and invasion. As described in the main text, TRPM3 and TRPV2-related drugs are closely associated with the central pore. However, whether TRPM3-related drugs can also promote the absorption of chemotherapeutic drugs through the intermediate pore, future studies should focus on these aspects.

With the increasing understanding of TRPM channel-mediated signaling pathways in tumors, many therapeutic targets involved in glioma cell proliferation, self-renewal, invasion, and angiogenesis have been identified. As a result, many novel treatments have been reported in the past decade. Few of these significantly affect glioma, even though anti-angiogenic therapy may provide some benefits for GBM patients, such as a possible delay in recurrence of about six months. Therefore, there is a need to develop other molecular targets that can bind to anti-angiogenic, cytotoxic, immunotherapy, and DNA repair inhibitors. In this case, targeting the activity of cytokines or components in the tumor microenvironment is also promising.

TRPM channels are overexpressed in tumors, including gliomas. Tumor microenvironments include a variety of cell types. Current studies are limited to tumor cells, so the cell types expressing TRPM channels in tumor microenvironments need to be further explored. TRPM channels are not universally expressed and may play a pleiotropic role. For example, TRPM channels are involved in vascular endothelial growth factor (VEGF) signaling, which influences angiogenesis and tumor growth. TRPM channels have also been demonstrated to interact with other channel families, such as the BK channels, which are directly involved in the signaling pathway of brain tumor progression. Furthermore, some researchers strongly support the use of TRPMs in antiangiogenic therapy. Since TRPM2 and TRPM7 are also found in cerebral blood vessels, it is suggested that they may promote GBM angiogenesis. Moreover, TRPM8 isoforms and TRPM2-AS may be more limited in nonmalignant human organs and thus may represent more specific targets. Certain studies did not measure intracellular ion concentrations or changes in ion fluxes that modulate the activity of specific TRPM channels, nor did they measure changes in specific TRPM currents. Therefore, further experiments are needed to discover the specific mechanisms.

Finally, in the last 3–4 years, significant discoveries have been made in the structure resolution of TRPM2, TRPM7, and TRPM8 by cryogenic EM. Due to the advances of cryo-EM methods in resolving complex receptor or ion channel structures embedded in plasma membranes, it is conceivable that we will gradually discover the structure of other TRPM channels. These data will aid in designing drugs with precise action and specific inhibitory activity, thereby expanding the potential of targeting TRPM pathways. TRPM channels can destroy human cancer cells, as shown by various evidence of their carcinogenic properties. In addition, future in vivo and in vitro evaluations and human clinical trials are needed to fully understand the role of TRPM ion channels in tumors such as gliomas. In short, the pharmaceutical industry and medical practice should consider them as new targets for therapeutic approaches.

## Abbreviations

CNS: central nervous system
TRP: transient receptor potential
TRPM: transient receptor potential melastatin
cADPR: cyclic ADPR
NAD: nicotinamide adenine dinucleotide
NAAD: nicotinate adenine dinucleotide
NAADP: NAAD-phosphate
RNS: reactive nitrogen
MAPKs: mitogen-activated protein kinases
PKC: protein kinase C
PLC: phospholipase C
ER: endoplasmic reticulum
GBM: glioblastoma multiforme
Se: selenium
miRNAs: MicroRNAs
CSCs: cancer stem cells
MRE: miRNA recognition element
EMT: epithelial-to-mesenchymal transformation
PS: pregnenolone sulfate
ALDH1: aldehyde dehydrogenase 1
GSC: glioma stem cell
CaMKII: Ca2+/calmodulin-dependent protein kinase II
LSCC: laryngeal squamous cell carcinoma
DTX: docetaxel
VQ-1: Vacquinol-1
VEGF: vascular endothelial growth factor
PIP2: phosphatidylinositol 4,5-bisphosphate
ADPR: ADP-ribose
CAM: calmodulin
TNFα: tumor necrosis factor-α
RNS: reactive nitrogen
PKC: protein kinase C
